# 2-(2-Thien­yl)-4,5-dihydro-1*H*-imidazole

**DOI:** 10.1107/S1600536809001068

**Published:** 2009-01-14

**Authors:** Reza Kia, Hoong-Kun Fun, Hadi Kargar

**Affiliations:** a X-ray Crystallography Unit, School of Physics, Universiti Sains Malaysia, 11800 USM, Penang, Malaysia; b Department of Chemistry, School of Science, Payame Noor University (PNU), Ardakan, Yazd, Iran

## Abstract

In title compound, C_7_H_8_N_2_S, the five-membered rings are twisted by a dihedral angle of 5.17 (10)°. Two inter­molecular N—H⋯N and C—H⋯N hydrogen bonds to the same acceptor N atom form seven-membered rings, producing *R*_2_^1^(7) ring motifs. These inter­actions link neighbouring mol­ecules into one-dimensional chains extended along the *c* axis. The crystal structure is further stabilized by weak inter­molecular C—H⋯π inter­actions.

## Related literature

For reference geometrical data, see: Allen *et al.* (1987 ▶). For details of hydrogen-bond motifs, see: Bernstein *et al.* (1995 ▶). For a related structure and the synthesis, see, Kia *et al.* (2008 ▶); Stibrany *et al.* (2004 ▶). For the applications of imidazoline derivatives, see, for example: Blancafort (1978 ▶); Chan (1993 ▶); Vizi (1986 ▶); Li *et al.* (1996 ▶); Ueno *et al.* (1995 ▶); Corey & Grogan (1999 ▶).
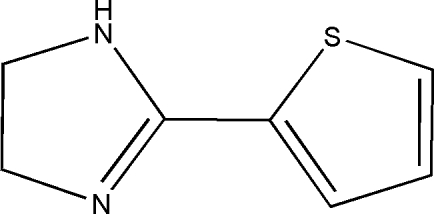


## Experimental

### 

#### Crystal data

C_7_H_8_N_2_S
*M* *_r_* = 152.21
Monoclinic, 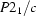

*a* = 6.1321 (2) Å
*b* = 11.5663 (3) Å
*c* = 10.0098 (3) Å
β = 90.154 (1)°
*V* = 709.95 (4) Å^3^
*Z* = 4
Mo *K*α radiation
μ = 0.37 mm^−1^
*T* = 100.0 (1) K
0.54 × 0.28 × 0.22 mm

#### Data collection

Bruker SMART APEXII CCD area-detector diffractometer
Absorption correction: multi-scan (**SADABS**; Bruker, 2005 ▶) *T*_min_ = 0.825, *T*_max_ = 0.922
27675 measured reflections
3100 independent reflections
3040 reflections with *I* > 2σ(*I*)
*R*_int_ = 0.021

#### Refinement

*R*[*F* ^2^ > 2σ(*F* ^2^)] = 0.050
*wR*(*F* ^2^) = 0.128
*S* = 1.24
3100 reflections
91 parameters
H-atom parameters constrained
Δρ_max_ = 0.62 e Å^−3^
Δρ_min_ = −0.39 e Å^−3^

### 

Data collection: *APEX2* (Bruker, 2005 ▶); cell refinement: *SAINT* (Bruker, 2005 ▶); data reduction: *SAINT*; program(s) used to solve structure: *SHELXTL* (Sheldrick, 2008 ▶); program(s) used to refine structure: *SHELXTL*; molecular graphics: *SHELXTL*; software used to prepare material for publication: *SHELXTL* and *PLATON* (Spek, 2003 ▶).

## Supplementary Material

Crystal structure: contains datablocks global, I. DOI: 10.1107/S1600536809001068/at2706sup1.cif

Structure factors: contains datablocks I. DOI: 10.1107/S1600536809001068/at2706Isup2.hkl

Additional supplementary materials:  crystallographic information; 3D view; checkCIF report

## Figures and Tables

**Table 1:** Hydrogen-bond geometry (Å, °)

*D*—H⋯*A*	*D*—H	H⋯*A*	*D*⋯*A*	*D*—H⋯*A*
N1—H1*N*1⋯N2^i^	0.75	2.23	2.977 (2)	171
C3—H3*A*⋯N2^i^	0.95	2.60	3.482 (2)	155
C6—H6*A*⋯*Cg*1^ii^	0.99	2.89	3.539 (2)	124
C6—H6*B*⋯*Cg*1^iii^	0.99	2.83	3.691 (2)	146

Symmetry codes: (i) 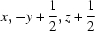; (ii) 
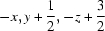
; (iii) 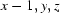. *Cg*1 is the centroid of the S1/C1–C4 (thiophen) ring.

**Fig. 1.**
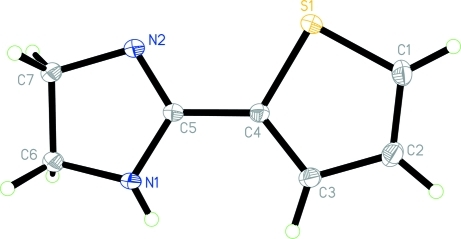
The molecular structure of (I) with atom labels and 50% probability ellipsoids for non-H atoms.

**Fig. 2.**
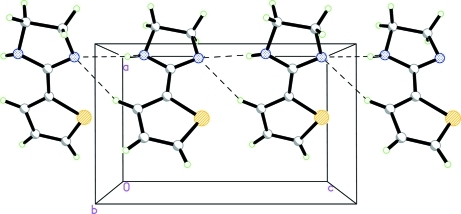
The crystal packing of (I), viewed down the b-axis showing 1-D infinite chain along the c-axis by intermolecular N—H···N and C—H···N interactions. The intermolecular interactions are shown as dashed lines.

## supplementary crystallographic information

### Comment

Imidazoline derivatives are of great importance because they exhibit significant biological and pharmacological activities including antihypertensive (Blancafort 1978), antihyperglycemic (Chan 1993), antidepressive (Vizi 1986), antihypercholesterolemic (Li *et al.*, 1996) and antiinflammatory properties (Ueno *et al.*, 1995). These compounds are also used as catalysts and synthetic intermediates in some organic reactions (Corey & Grogan 1999). Due to these important applications of imidazolines, here we report the crystal structure of the title compound (I).

In the title compound (I) (Fig. 1), bond lengths (Allen *et al.* 1987) and angles are within the normal ranges and are comparable with the related structures (Stibrany *et al.* 2004; Kia *et al.*, 2008). The two five-membered rings are not coplanar to each other and twisted by a dihedral angle of 5.17 (10)°. Two intermolecular N—H···N and C—H···N hydrogen bonds involving a nitrogen atom as an acceptor form seven-membered rings, producing, *R^1^_2_(7)* ring motifs (Table 1). These interactions link neighbouring molecules into 1-D extended chains along the *c* axis (, Fig. 2). The crystal structure is further stabilized by weak intermolecular C—H···π interactions [C6—H6A···*Cg*1^i^ and C6—H6B···*Cg*1^ii^: (i) -x, 1/2 + y, 3/2 - z, (ii) -1 + x, y, z; *Cg*1 is the centroid of the S1/C1–C4 thiophene ring.

### Experimental

The synthetic method was based on the previous work (Stibrany *et al.* 2004), except that 10 mmol of 2-cyano-thiophene and 40 mmol of ethylenediamine was used. Single crystals suitable for *X*-ray diffraction were obtained by evaporation of a toluene solution at room temperature.

### Refinement

The hydrogen atom bound to N1 was located from the difference Fourier map and constrained to refine with the respective parent atom, see Table 1. The rest of the hydrogen atoms were positioned gemetrically and refined in a riding model approximation with C—H = 0.95–0.99 Å and *U*_iso_ (H) = 1.2 *U*_eq_ (C).

### Crystal data

**Table:**

C_7_H_8_N_2_S	*F*(000) = 320
*M**_r_* = 152.21	*D*_x_ = 1.424 Mg m^−^^3^
Monoclinic, *P*2_1_/*c*	Mo *K*α radiation, λ = 0.71073 Å
Hall symbol: -P 2ybc	Cell parameters from 9869 reflections
*a* = 6.1321 (2) Å	θ = 2.5–34.3°
*b* = 11.5663 (3) Å	µ = 0.37 mm^−^^1^
*c* = 10.0098 (3) Å	*T* = 100 K
β = 90.154 (1)°	Block, colourless
*V* = 709.95 (4) Å^3^	0.54 × 0.28 × 0.22 mm
*Z* = 4	

### Data collection

**Table:**

Bruker SMART APEXII CCD area-detector diffractometer	3100 independent reflections
Radiation source: fine-focus sealed tube	3040 reflections with *I* > 2σ(*I*)
graphite	*R*_int_ = 0.021
φ and ω scans	θ_max_ = 35.0°, θ_min_ = 2.7°
Absorption correction: multi-scan (*SADABS*; Bruker, 2005)	*h* = −9→9
*T*_min_ = 0.825, *T*_max_ = 0.922	*k* = −18→17
27675 measured reflections	*l* = −15→15

### Refinement

**Table:**

Refinement on *F*^2^	Primary atom site location: structure-invariant direct methods
Least-squares matrix: full	Secondary atom site location: difference Fourier map
*R*[*F*^2^ > 2σ(*F*^2^)] = 0.050	Hydrogen site location: inferred from neighbouring sites
*wR*(*F*^2^) = 0.128	H-atom parameters constrained
*S* = 1.24	*w* = 1/[σ^2^(*F*_o_^2^) + 1.7551*P*] where *P* = (*F*_o_^2^ + 2*F*_c_^2^)/3
3100 reflections	(Δ/σ)_max_ < 0.001
91 parameters	Δρ_max_ = 0.62 e Å^−^^3^
0 restraints	Δρ_min_ = −0.39 e Å^−^^3^

### Special details

**Table:**

Experimental. The low-temperature data was collected with the Oxford Cyrosystem Cobra low-temperature attachment.
Geometry. All e.s.d.'s (except the e.s.d. in the dihedral angle between two l.s. planes) are estimated using the full covariance matrix. The cell e.s.d.'s are taken into account individually in the estimation of e.s.d.'s in distances, angles and torsion angles; correlations between e.s.d.'s in cell parameters are only used when they are defined by crystal symmetry. An approximate (isotropic) treatment of cell e.s.d.'s is used for estimating e.s.d.'s involving l.s. planes.
Refinement. Refinement of *F*^2^ against ALL reflections. The weighted *R*-factor *wR* and goodness of fit *S* are based on *F*^2^, conventional *R*-factors *R* are based on *F*, with *F* set to zero for negative *F*^2^. The threshold expression of *F*^2^ > σ(*F*^2^) is used only for calculating *R*-factors(gt) *etc*. and is not relevant to the choice of reflections for refinement. *R*-factors based on *F*^2^ are statistically about twice as large as those based on *F*, and *R*- factors based on ALL data will be even larger.

### Fractional atomic coordinates and isotropic or equivalent isotropic displacement parameters (Å^2^)

**Table:**

	*x*	*y*	*z*	*U*_iso_*/*U*_eq_	
S1	0.46967 (8)	0.14161 (4)	0.59514 (4)	0.01548 (10)	
N2	0.0669 (2)	0.29635 (14)	0.61122 (15)	0.0137 (2)	
N1	0.0460 (2)	0.30376 (14)	0.83746 (14)	0.0133 (2)	
H1N1	0.0584	0.2724	0.9026	0.016*	
C1	0.6387 (3)	0.05389 (17)	0.6858 (2)	0.0180 (3)	
H1A	0.7542	0.0100	0.6482	0.022*	
C2	0.5888 (3)	0.05418 (16)	0.81838 (19)	0.0169 (3)	
H2A	0.6655	0.0103	0.8835	0.020*	
C3	0.4092 (3)	0.12742 (15)	0.84860 (17)	0.0136 (3)	
H3A	0.3530	0.1385	0.9360	0.016*	
C4	0.3260 (3)	0.18060 (14)	0.73589 (16)	0.0117 (3)	
C5	0.1427 (3)	0.26052 (14)	0.72556 (16)	0.0109 (3)	
C6	−0.1543 (3)	0.36276 (17)	0.79447 (17)	0.0154 (3)	
H6A	−0.1724	0.4380	0.8403	0.018*	
H6B	−0.2849	0.3144	0.8101	0.018*	
C7	−0.1097 (3)	0.37874 (16)	0.64419 (17)	0.0151 (3)	
H7A	−0.2423	0.3615	0.5912	0.018*	
H7B	−0.0634	0.4591	0.6252	0.018*	

### Atomic displacement parameters (Å^2^)

**Table:**

	*U*^11^	*U*^22^	*U*^33^	*U*^12^	*U*^13^	*U*^23^
S1	0.01727 (19)	0.01847 (19)	0.01071 (17)	0.00446 (15)	0.00185 (13)	−0.00026 (14)
N2	0.0153 (6)	0.0161 (6)	0.0098 (6)	0.0027 (5)	0.0003 (4)	0.0009 (5)
N1	0.0148 (6)	0.0170 (6)	0.0080 (5)	0.0029 (5)	0.0016 (4)	0.0004 (5)
C1	0.0166 (7)	0.0181 (8)	0.0192 (8)	0.0057 (6)	−0.0001 (6)	−0.0015 (6)
C2	0.0186 (7)	0.0155 (7)	0.0167 (7)	0.0029 (6)	−0.0034 (6)	0.0009 (6)
C3	0.0164 (7)	0.0129 (7)	0.0113 (6)	−0.0001 (5)	−0.0002 (5)	0.0004 (5)
C4	0.0131 (6)	0.0120 (6)	0.0100 (6)	0.0005 (5)	−0.0003 (5)	−0.0005 (5)
C5	0.0120 (6)	0.0113 (6)	0.0094 (6)	−0.0011 (5)	0.0005 (5)	−0.0004 (5)
C6	0.0143 (7)	0.0186 (7)	0.0132 (7)	0.0030 (6)	0.0019 (5)	0.0007 (6)
C7	0.0152 (7)	0.0181 (7)	0.0120 (7)	0.0035 (6)	0.0000 (5)	0.0018 (5)

### Geometric parameters (Å, °)

**Table:**

S1—C1	1.7099 (19)	C2—H2A	0.9500
S1—C4	1.7239 (17)	C3—C4	1.381 (2)
N2—C5	1.302 (2)	C3—H3A	0.9500
N2—C7	1.480 (2)	C4—C5	1.459 (2)
N1—C5	1.364 (2)	C6—C7	1.541 (2)
N1—C6	1.468 (2)	C6—H6A	0.9900
N1—H1N1	0.7501	C6—H6B	0.9900
C1—C2	1.362 (3)	C7—H7A	0.9900
C1—H1A	0.9500	C7—H7B	0.9900
C2—C3	1.423 (3)		
			
C1—S1—C4	91.82 (9)	C5—C4—S1	120.22 (12)
C5—N2—C7	105.57 (14)	N2—C5—N1	116.78 (15)
C5—N1—C6	107.14 (14)	N2—C5—C4	122.49 (15)
C5—N1—H1N1	119.6	N1—C5—C4	120.71 (14)
C6—N1—H1N1	124.2	N1—C6—C7	101.03 (13)
C2—C1—S1	112.21 (14)	N1—C6—H6A	111.6
C2—C1—H1A	123.9	C7—C6—H6A	111.6
S1—C1—H1A	123.9	N1—C6—H6B	111.6
C1—C2—C3	112.63 (16)	C7—C6—H6B	111.6
C1—C2—H2A	123.7	H6A—C6—H6B	109.4
C3—C2—H2A	123.7	N2—C7—C6	105.80 (14)
C4—C3—C2	112.08 (15)	N2—C7—H7A	110.6
C4—C3—H3A	124.0	C6—C7—H7A	110.6
C2—C3—H3A	124.0	N2—C7—H7B	110.6
C3—C4—C5	128.52 (15)	C6—C7—H7B	110.6
C3—C4—S1	111.26 (13)	H7A—C7—H7B	108.7
			
C4—S1—C1—C2	−0.16 (16)	C6—N1—C5—N2	11.9 (2)
S1—C1—C2—C3	−0.2 (2)	C6—N1—C5—C4	−169.73 (15)
C1—C2—C3—C4	0.5 (2)	C3—C4—C5—N2	−173.27 (18)
C2—C3—C4—C5	179.52 (17)	S1—C4—C5—N2	6.9 (2)
C2—C3—C4—S1	−0.60 (19)	C3—C4—C5—N1	8.5 (3)
C1—S1—C4—C3	0.44 (14)	S1—C4—C5—N1	−171.36 (13)
C1—S1—C4—C5	−179.67 (15)	C5—N1—C6—C7	−17.79 (18)
C7—N2—C5—N1	0.6 (2)	C5—N2—C7—C6	−12.26 (19)
C7—N2—C5—C4	−177.67 (15)	N1—C6—C7—N2	18.12 (18)

### Hydrogen-bond geometry (Å, °)

**Table:**

*D*—H···*A*	*D*—H	H···*A*	*D*···*A*	*D*—H···*A*
N1—H1N1···N2^i^	0.75	2.23	2.977 (2)	171
C3—H3A···N2^i^	0.95	2.60	3.482 (2)	155
C6—H6A···Cg1^ii^	0.99	2.89	3.539 (2)	124
C6—H6B···Cg1^iii^	0.99	2.83	3.691 (2)	146

Symmetry codes: (i) *x*, −*y*+1/2, *z*+1/2; (ii) −*x*, *y*+1/2, −*z*+3/2; (iii) *x*−1, *y*, *z*.
