# 2-(2-Thien­yl)-4,5-dihydro-1*H*-imidazole. Corrigendum

**DOI:** 10.1107/S1600536809018790

**Published:** 2009-05-23

**Authors:** Reza Kia, Hoong-Kun Fun, Hadi Kargar

**Affiliations:** a X-ray Crystallography Unit, School of Physics, Universiti Sains Malaysia, 11800 USM, Penang, Malaysia; b Department of Chemistry, School of Science, Payame Noor University (PNU), Ardakan, Yazd, Iran

## Abstract

Consideration of a previous unrecognized twinning of the original investigated crystal of the title compound [Kia *et al.* (2009 ▶). *Acta Cryst.* E**65**, o301] led to improved reliability factors and to a slightly higher precision for all geometric parameters. The crystal under investigation was twinned by pseudo-merohedry with [100, 00, 00] as the twin matrix and a refined twin domain fraction of 0.9610 (5):0.0390 (5). The results of the new crystal structure refinement are given here.

## Abstract 2

Corrigendum to *Acta Cryst.* (2009), E**65**, o301.

## Experimental

### 

#### Crystal data

C_7_H_8_N_2_S
*M* *_r_* = 152.21
Monoclinic, 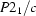

*a* = 6.1321 (2) Å
*b* = 11.5663 (3) Å
*c* = 10.0098 (3) Å
β = 90.154 (1)°
*V* = 709.95 (4) Å^3^
*Z* = 4
Mo *K*α radiation
μ = 0.37 mm^−1^
*T* = 100 K
0.54 × 0.28 × 0.22 mm

#### Data collection

Bruker SMART APEXII CCD area-detector diffractometer
Absorption correction: multi-scan (**SADABS**; Bruker, 2005 ▶) *T*_min_ = 0.825, *T*_max_ = 0.922
28316 measured reflections
3100 independent reflections
3040 reflections with *I* > 2σ(*I*)
*R*_int_ = 0.021

#### Refinement

*R*[*F* ^2^ > 2σ(*F* ^2^)] = 0.027
*wR*(*F* ^2^) = 0.080
*S* = 1.15
3100 reflections
96 parameters
H atoms treated by a mixture of independent and constrained refinement
Δρ_max_ = 0.52 e Å^−3^
Δρ_min_ = −0.24 e Å^−3^

### 

Data collection: *APEX2* (Bruker, 2005 ▶); cell refinement: *SAINT* (Bruker, 2005 ▶); data reduction: *SAINT*; program(s) used to solve structure: *SHELXTL* (Sheldrick, 2008 ▶); program(s) used to refine structure: *SHELXTL* (Sheldrick, 2008 ▶); molecular graphics: *SHELXTL* (Sheldrick, 2008 ▶); software used to prepare material for publication: *SHELXTL* (Sheldrick, 2008 ▶) and *PLATON* (Spek, 2009 ▶).

## Supplementary Material

Crystal structure: contains datablocks global, I. DOI: 10.1107/S1600536809018790/wm2233sup1.cif

Structure factors: contains datablocks I. DOI: 10.1107/S1600536809018790/wm2233Isup2.hkl

. DOI: 10.1107/S1600536809018790/wm2233fig1.tif
The mol­ecular structure of the title compound showing the atom labels and 50% probability displacement ellipsoids for non-H atoms.

*a* *c*. DOI: 10.1107/S1600536809018790/wm2233fig2.tif
The crystal packing of the title compound, viewed down the *a* axis, showing the one-dimensional infinite chain along the *c* axis formed by inter­molecular N—H⋯N and C—H⋯N inter­actions. The inter­molecular inter­actions are shown as dashed lines.

Additional supplementary materials:  crystallographic information; 3D view; checkCIF report

## Figures and Tables

**Table 1:** Hydrogen-bond geometry (Å, °)

*D*—H⋯*A*	*D*—H	H⋯*A*	*D*⋯*A*	*D*—H⋯*A*
N1—H1⋯N2^i^	0.857 (16)	2.130 (16)	2.9803 (10)	171.5 (16)
C3—H3*A*⋯N2^i^	0.95	2.59	3.4815 (11)	156

Symmetry code: (i) 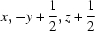.
